# Association Between Awareness of Hypertension and Health-Related Quality of Life in a Cross-Sectional Population-Based Study in Rural Area of Northwest China

**DOI:** 10.1097/MD.0000000000001206

**Published:** 2015-07-24

**Authors:** Baibing Mi, Shaonong Dang, Qiang Li, Yaling Zhao, Ruihai Yang, Duolao Wang, Hong Yan

**Affiliations:** From the Department of Epidemiology and Biostatistics, School of Public Health, Xi’an Jiaotong University Health Science Center, Xi’an (BM, SD, QL, YZ, HY); Department of Cardiovascular Diseases, Hanzhong People's Hospital, Hanzhong, Shaanxi, People's Republic of China (RY); and Department of Clinical Sciences, Liverpool School of Tropical Medicine Pembroke Place, Liverpool, L3 5QA, UK (DW)

## Abstract

Hypertensive patients have more complex health care needs and are more likely to have poorer health-related quality of life than normotensive people. The awareness of hypertension could be related to reduce health-related quality of life. We propose the use of quantile regression to explore more detailed relationships between awareness of hypertension and health-related quality of life.

In a cross-sectional, population-based study, 2737 participants (including 1035 hypertensive patients and 1702 normotensive participants) completed the Short-Form Health Survey. A quantile regression model was employed to investigate the association of physical component summary scores and mental component summary scores with awareness of hypertension and to evaluate the associated factors.

Patients who were aware of hypertension (N = 554) had lower scores than patients who were unaware of hypertension (N = 481). The median (IQR) of physical component summary scores: 48.20 (13.88) versus 53.27 (10.79), *P* < 0.01; the mental component summary scores: 50.68 (15.09) versus 51.70 (10.65), *P* = 0.03. adjusting for covariates, the quantile regression results suggest awareness of hypertension was associated with most physical component summary scores quantiles (*P* < 0.05 except 10th and 20th quantiles) in which the *β*-estimates from −2.14 (95% CI: −3.80 to −0.48) to −1.45 (95% CI: −2.42 to −0.47), as the same significant trend with some poorer mental component summary scores quantiles in which the *β*-estimates from −3.47 (95% CI: −6.65 to −0.39) to −2.18 (95% CI: −4.30 to −0.06). The awareness of hypertension has a greater effect on those with intermediate physical component summary status: the *β*-estimates were equal to −2.04 (95% CI: −3.51 to −0.57, *P* < 0.05) at the 40th and decreased further to −1.45 (95% CI: −2.42 to −0.47, *P* < 0.01) at the 90th quantile.

Awareness of hypertension was negatively related to health-related quality of life in hypertensive patients in rural western China, which has a greater effect on mental component summary scores with the poorer status and on physical component summary scores with the intermediate status.

## INTRODUCTION

Hypertension is known as a risk factor for cardio-cerebrovascular disease,^1^ and places a heavy burden on individuals, families, and society.^2^ Hypertensive patients have more complex health care needs and are more likely to have poorer health-related quality of life (HRQoL) than normotensive people.^3–6^ Given China's rapid socioeconomic development, the prevalence of hypertension is increasing, especially among elderly people.^7^ The management of hypertension pose challenges to health care providers.

Given the reduction in the long-term risk of cardio-cerebrovascular morbidity and mortality, it is imperative to evaluate the effect of hypertension on HRQoL. Some studies illustrated that hypertension causes serious damage to HRQoL.^8^ A recent systematic review of 20 observational studies concluded that hypertension reduces HRQoL but the magnitude is small.^9^

One speculative reason for hypertensive patients’ poorer HRQoL is their awareness of their hypertensive status; several studies have supported this hypothesis.^8,10,11^ However, these studies have commonly used multiple linear regression or logistic regression to model the measures of HRQoL. These methods are useful for interpreting the change in the mean of the conditional distribution of HRQoL, but may mask some of the important associations in various segments of the outcome distribution.^12^ By contrast, quantile regression offers an innovative and more concise means of capturing these effects in this area of research.^13^ In the present study, we propose the use of quantile regression to examine the relationship between hypertensive patients’ awareness of their hypertension and HRQoL while controlling for socioeconomic conditions, lifestyle factors, other potentially confounding factors and blood pressure.

## METHODS

### Study Design and Participants

This cross-sectional, community-based survey was carried out in the Hantai district of Hanzhong city in the province of Shaanxi, China. There were approximately 55,000 inhabitants in the study area, and more than 90% of them were farmers. The prevalence of hypertension in adults in rural areas is 34.3%. The sampling method of this survey has been described in detailed elsewhere.^14^

This survey was conducted in 2010. Trained professional interviewers from the Faculty of Public Health of Xi’an Jiaotong University interviewed the participants regarding their demographic and lifestyle characteristics, history of disease, and HRQoL. Blood pressure, height, and weight were measured by trained doctors and nurses from Hanzhong People's Hospital during a physical examination.

A total of 117 participants were excluded due to missing or implausible data, including persons who did not have a blood pressure measurement (n = 19) and those who did not complete the SF-36 HRQoL scales (n = 98). A total of 2737 participants were included in this analysis. This sample size was sufficient to detect a difference of 10 points in the SF-36 component summary scores between hypertensive patients who were aware of their hypertension and those who were not aware of their hypertension given *α* = 0.05 and *β* = 0.20.

### Assessment of Health-Related Quality of Life

To evaluate the effects of awareness of hypertension on HRQoL, the present study used the SF-36, whose validity and reliability have been tested in heterogeneous populations and hypertensive patients.^6^ This measure is a brief self-administered questionnaire that generates assessment scores across 36 scales;^15,16^ these scales are scored from 0 (poorest health) to 100 (optimal health). The following 8 dimensions of HRQoL are evaluated by these scores: physical function (PF), role limitations due to physical health condition (RP), bodily pain (BP), general health condition (GH), vitality (VT), social function status (SF), role limitations due to emotional health condition (RE), and mental health (MH). In addition, the instrument has a 1-item scale on health transition. The SF-36 dimensions can be reduced to 2 aggregate summaries, a physical component summary (PCS) and a mental component summary (MCS), which represent physical functioning condition and emotional condition, respectively.^17^ This scale's reliability and validity are widely documented across a range of language versions.^16^ The mandarin version of the SF-36 used in this study has been administered successfully in general populations in China^18–21^ and in populations with specific chronic diseases.^21–23^

### Assessment of Blood Pressure and Definitions

On the day before the medical examination, the participants were informed by the village doctors that they should fast for at least 8 hours and avoid heavy physical activity. Blood pressure was measured by trained health workers according to the standard operating procedures.^24^ This measure method according to a criterion formulated by the Chinese Hypertension League/National Center for Cardiovascular Disease (CHL/NCCD),^24^ hypertension was defined as systolic blood pressure (SBP) ≥140 mm Hg and/or diastolic blood pressure (DBP) ≥90 mm Hg or the use of antihypertensive medications.

The hypertensive subjects were identified as aware of their hypertensive status if they reported ever having been diagnosed with high blood pressure or hypertension by a health care provider. With this identification, these participants were classified into the following 2 groups: hypertensive patients with awareness of their hypertension (hereafter referred to as “aware of hypertension”) and patients without awareness of their hypertension (hereafter referred to as “unaware of hypertension”).

### Other Anthropometric Variables and Covariates

Body weight and height were collected by trained health workers according to standard operating procedures. Body mass index (BMI) was calculated as weight in kilograms divided by height in meters squared. Standardized interview questionnaire was a useful tool for collecting information on demographics (social-economic factors, family history of hypertension, total monthly family income and expenses, and so on), lifestyle (alcohol intake and cigarette smoking), comorbidities (cardiovascular disease, cerebrovascular disease, and diabetes), and anti-hypertensive medication that could influence the SF-36 questionnaire scores.^25^ Education stage was represented as the total years of schooling education, which was classified into primary school (≤6 years of schooling), secondary school (6–9 years of schooling), and senior school and higher education (≥9 years of schooling). Family history of hypertension was defined as someone in family (a blood relative such as mother, father, sister, or brother) who has or had hypertension. Participant's comorbidities were determined when they answered that they had been diagnosed by doctors with the condition. Anti-hypertensive medication was reported by themselves whether they used anti-hypertensive drugs or not. Persons who had never smoked or ever smoked fewer than 6 months were defined as never smokers. Those who did not consume alcoholic beverages (wine or beer) or ever consumed less than 1 cup per month were defined as nondrinkers.

### Statistical Analysis

All questionnaires were coded and double-entered using Epidata version 3.1 (the Epidata association, Odense, Denmark) by 2 independent data entry staff. To identify the family economic status of the participants, a wealth index based on communication tools, transportation tools, sources of water, and total monthly family income and expenses was established using principal component analysis. The first principal component was selected as the wealth index, and the participants’ family economic level was categorized into 3 groups, poor, moderate, and rich, according to the tertiles of the wealth index.^26^

Initially, the descriptive data on characteristics of the participants were summarized using mean ± SD and median with interquartile range (IQR) for normally abnormally distributed continuous variables, respectively. Count and proportions were used for descripting categorical variables. The *χ*^2^ statistical test was used to analyze differences in proportions among groups. The ANOVA (normally distributed data) or Kruskal–Wallis test (abnormally distributed data) was used to analyze differences in continuous variables among groups.

The distribution of the 8 dimensions and 2 component summary scores of the SF-36 was positively skewed,^27^ and ceiling and flooring effects were evident (Table 1). This result indicates that the true variation in some dimensions of HRQoL among those scoring 100 or 0 scores may not adequately be captured by traditional regression analysis.^28^

**TABLE 1:**
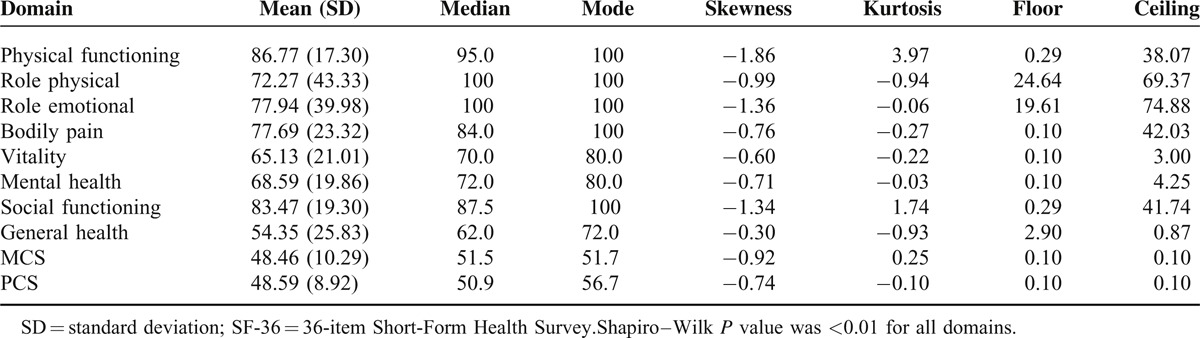
Descriptive Statistics of Each Domain of the SF-36^∗^

To address the skewed distribution of the SF-36 in our study for detecting the association between awareness of hypertension and HRQoL, we used quantile regression (QR) to estimate and conduct the inference about those conditional quantile functions.^13^ This method offers a mechanism for estimating the conditional quantile of the distribution of SF-36 scores. Unlike traditional linear regression based on the ordinary least square (OLS), QR is not limited to explaining the mean of the SF-36. Rather, it could also be used to explain the determinants of the SF-36 at any point on its distribution.

Therefore, the relationships among awareness of hypertension, other socio-demographic covariates, and HRQoL were examined using the multivariate QR model. The choice of percentiles largely depends on the distribution of the outcome and the research aims. Using SF-36 utility scores, 9 quantiles of HRQoL were selected (10th to 90th, step by 10th) from poorest to highest HRQoL. The association between awareness of hypertension and HRQoL was explored at poor (10th, 20th, 30th), intermediate (40th, 50th, 60th), and good (70th, 80th, 90th) quantiles.

Regression coefficients for each quantile and its 95% confidence interval (95% CI) were computed for aware of hypertension using not aware of hypertension as a reference. Five adjusted models containing these covariates were established step by step for each quantile to control for these cofounders. Model 1 adjusted for age and sex. Model 2 adjusted for the variables in model 1 plus some other demographic and lifestyle characteristics, including education, marital status, and wealth index, family history of hypertension, alcohol intake, and smoking. Model 3 adjusted for the variables in model 2 and for BMI and blood pressure (including both SBP and DBP). Model 4 additionally adjusted for the variables in model 3 plus some comorbidities (cardiovascular disease, cerebrovascular disease, and diabetes) to control the effect of comorbidities on HRQoL. Furthermore, model 5 adjusted for all the variables in model 4 plus anti-hypertensive medication.

All statistical analyses were performed using SAS 9.3 (SAS Institute Inc., Cary, NC). Two-tailed *P* < 0.05 was considered statistically significant.

### Ethics Statement

The study complied with the Declaration of Helsinki and was reviewed and approved by the Ethics Committee of Xi’an Jiaotong University Health Science Center. Furthermore, written informed consent was obtained from the study participants.

## RESULTS

### Characteristics of the Participants

In total, 1035 hypertensive patients and 1702 normotensive participants were evaluated. The respondents’ age ranged from 18 to 77 years, with a mean age of 50.11 years. Of the participants, 481 (17.57%) were not aware and 554 (20.24%) were aware of hypertension. Demographics, lifestyle factors, and comorbidities were significantly different among these 3 groups (Table 2). Compared with normotensive and unawareness of hypertension groups, patients in awareness of hypertension group were older (57.06 ± 9.57 years), had higher blood pressure levels (SBP: 159.19 ± 21.48 mm Hg, DBP: 89.23 ± 11.45 mm Hg), higher mean BMI index (24.25 ± 3.24), and worse educated (primary school or lower: n = 323, 58.62%) and poorer (n = 107, 19.31%), and these differences were statistically significant.

**TABLE 2:**
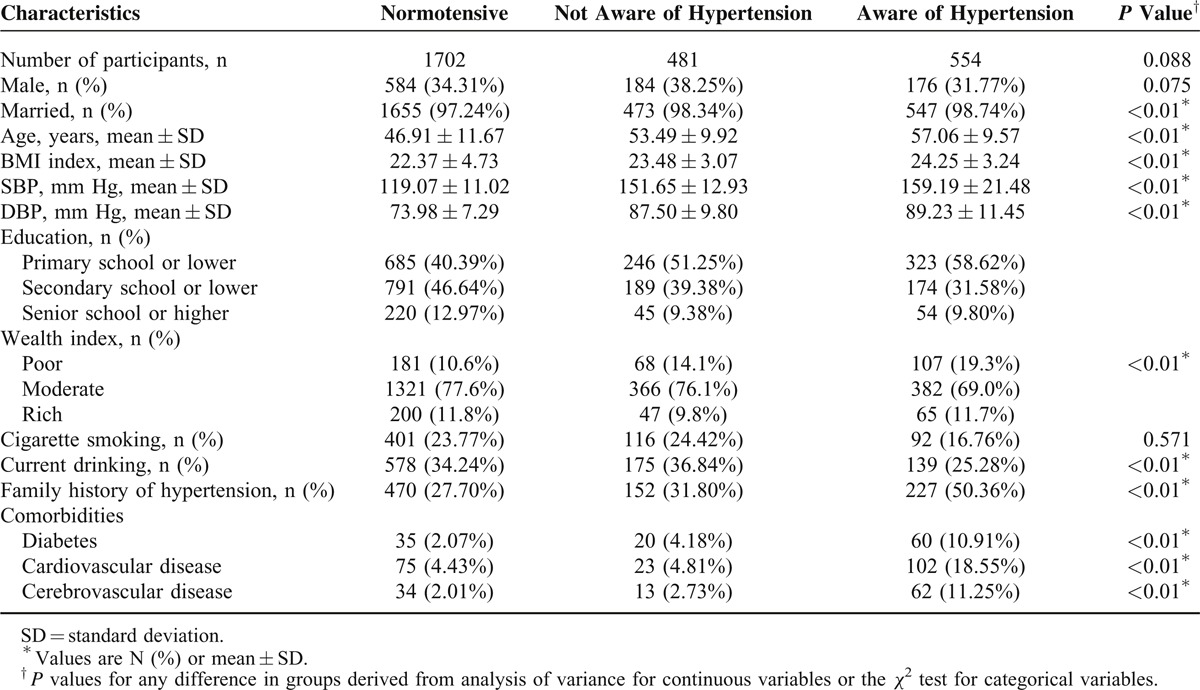
Sociodemographic Characteristics of 1702 Normotensive and 1035 Hypertensive Patients in Rural Hanzhong (N (%) or Mean ± SD)^∗^

### Health-Related Quality of Life Among the Groups

**FIGURE 1:**
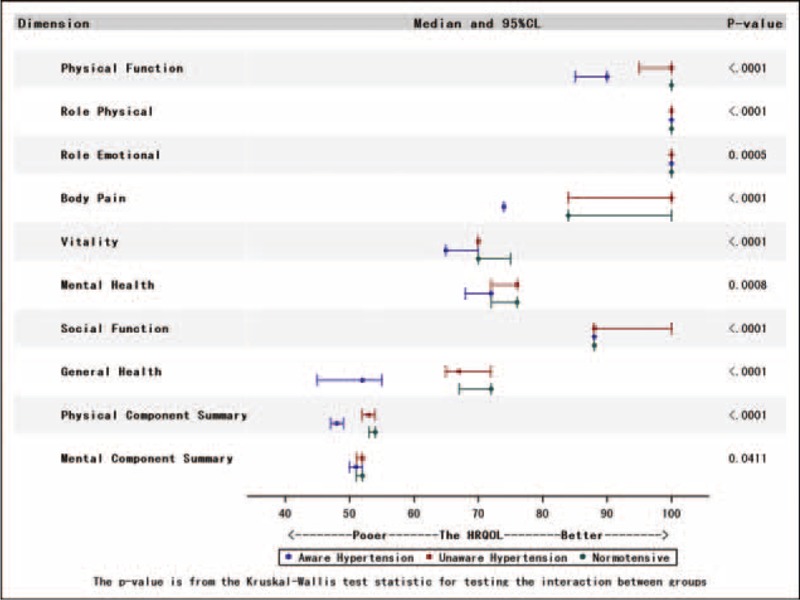
This figure illustrates the median and 95% confidence intervals of health-related quality of life dimensions according to normotensive and categories of awareness of hypertensive status.

The examination of HRQoL categories by hypertension status (aware of hypertension, unaware of hypertension, and normotensive) showed a statistically significant difference in some of the 8 dimension scales among the 3 groups, especially between aware and unaware of hypertension groups (Figure 1). Furthermore, the median (IQR) PCS score was 48.20 (13.88) for patients who were aware of hypertension and 53.27 (10.79) for patients who were not aware of hypertension (*P* < 0.01). The median (IQR) MCS score was 50.68 (15.09) for patients who were aware of hypertension and 51.70 (10.65) for those who were not aware of hypertension (*P* = 0.03). In the univariate analysis, patients who were aware of hypertension had lower scores than patients who were unaware of hypertension and normotensive.

### Association Between HRQoL and Awareness of Hypertension

In Table 3, the OLS and QR results obtained for MCS scores as an outcome variable were reported to represent the entire HRQoL. After sensitivity analysis adjusting for all variables from model 1 to model 3, the *β*-estimates showed that lower MCS scores were associated with awareness of hypertension. Specifically, a significant association between awareness of hypertension and the MCS scores was found among patients with poor HRQoL status.

**TABLE 3:**
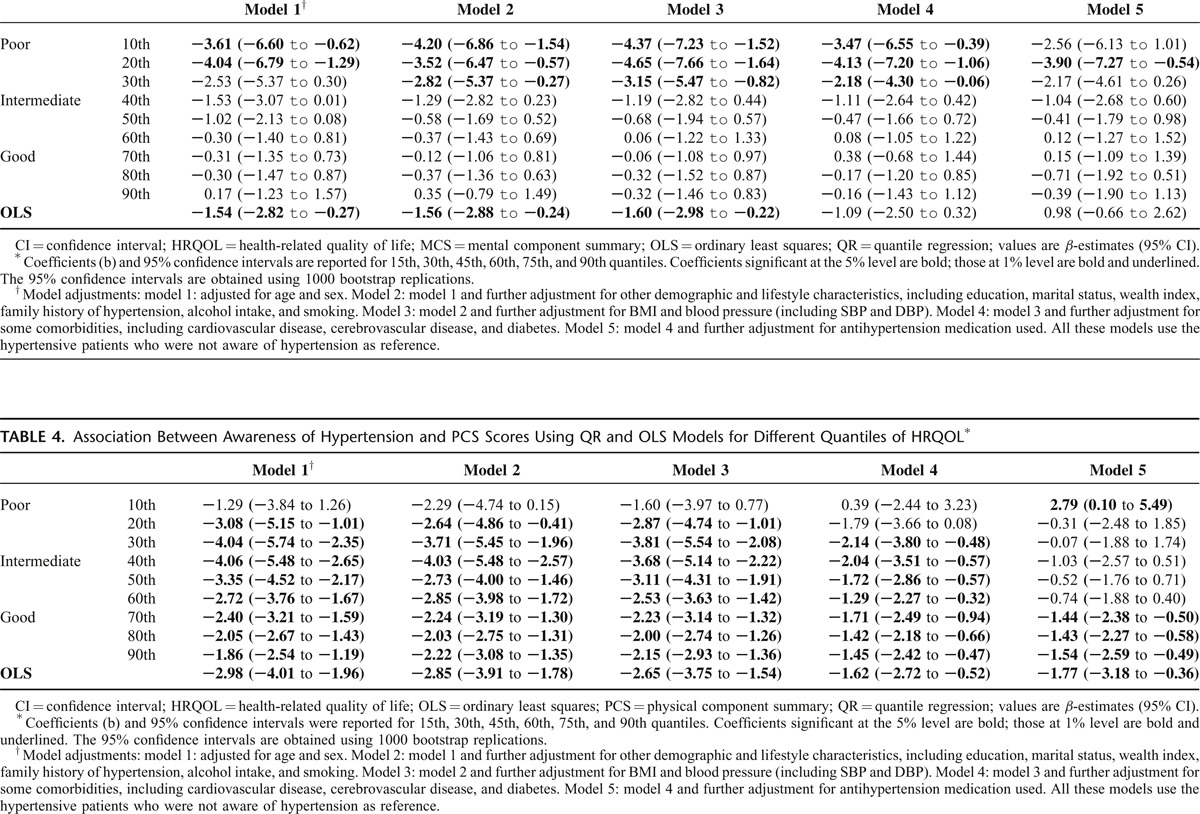
Association Between Awareness of Hypertension and MCS Scores Using QR and OLS Models for Different Quantiles of HRQOL^∗^

As shown in Table 4, the relationship that lower MCS scores were associated with awareness of hypertension is statistically significant and robust. Moreover, the effect of awareness of hypertension decreased as the location on the PCS score distribution changed from central (40th quantile) to tail (10th and 90th quantiles). This result confirms the conjecture of a location-scale model, namely, of changes both in the central tendency and in the variability of the PCS score. For example, in model 1, for those in the central location (in 30th, 40th, and 50th quantiles), awareness of hypertension led to a decrease in the PCS score by 4.04, 4.06, and 3.35, respectively. This effect was equal to 1.86 at the 90th and decreased further to 1.29 at the 10th quantile. In practical terms, the QR results suggest that awareness of hypertension has a greater effect on those with intermediate PCS status, that is, greater than or equal to the 40th quantile (*β* = 4.06). This trend was also found in other models (model 2 and model 3).

**TABLE 4:**
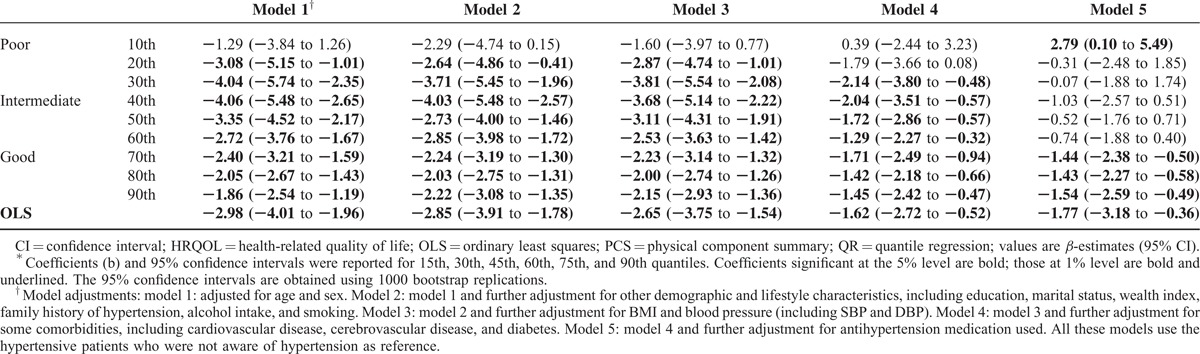
Association Between Awareness of Hypertension and PCS Scores Using QR and OLS Models for Different Quantiles of HRQOL^∗^

**FIGURE 2:**
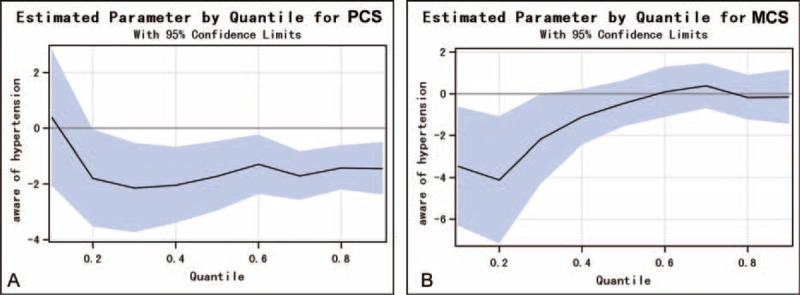
A, This figure clearly indicates that the regression coefficients varied at different quantiles of the PCS of the SF-36 after controlling for confounding factors. B, This figure clearly indicates that the regression coefficients varied at different quantiles of the MCS of the SF-36 after controlling for confounding factors.

Furthermore, we investigated whether awareness of hypertension had a significant impact on the 2 component summary scores after adjusting for comorbidities (model 4) and antihypertensive medication using (model 5). Adjustment for comorbidities led to a 40% reduction in the magnitude of the association between hypertension awareness and HRQoL at any quantile of the PCS scale (Table 4, model 4 vs. model 3). However, awareness of hypertension was still independently associated with most PCS quantiles (*P* < 0.05 except 10th and 20th quantiles, Figure 2A) and some MCS quantiles (*P* < 0.05 in poor HRQoL status, Figure 2B) even.

For antihypertensive medication, we found a considerable reduction (42–90%) in the magnitude of the association between hypertension awareness and HRQoL at poor and intermediate quantiles of the PCS scale (Table 4, model 5 vs. model 4). However, awareness of hypertension was still an independent factor which is associated with good PCS quantiles (*P* < 0.05 from 70th and 90th quantiles, Table 4). However, these significant influences between hypertension awareness and HRQoL were not observed at the MCS scale (Table 3, model 5 vs. model 4).

With these results, we can conclude that awareness of hypertension is negatively related to HRQoL in hypertensive patients, with a greater effect on MCS only with the poorer status and on PCS with the intermediate and good status. Even considering antihypertensive medication use, awareness of hypertension has a significantly negative influence on PCS scale, especially in good HRQoL status.

## DISCUSSION

### Main Findings and Their Significance

Our study found that awareness of hypertension was negatively associated with HRQoL in hypertensive patients who lived in rural western China. After adjusting for relevant socio-demographic, anthropometry, and lifestyle parameters and comorbidities, a lower PCS and MCS score was reported among those who were aware of their hypertension versus those who were not aware. Using the quantile regression modeling method, our results were generalized to the whole distribution of HRQoL and especially found that awareness of hypertension had a greater effect on MCS with the poorer status and on PCS with the intermediate and good status.

This present study supports the findings of earlier studies from Spain and Finland, which showed the negative association between awareness of hypertension and HRQoL.^10,29^ This result is consistent with other studies that found lower HRQoL scores among those aware that they had hypertension compared with those who were not aware or were normotensive.^8,30,31^ Our findings confirmed this association in rural western of China.

### Comparisons with Other Studies and Implications of Findings

The finding that patients who were aware of hypertension tend to report lower HRQoL has been found in Spain and Finland.^4,5,8,10,11^ The first population-based study on the impact of awareness of hypertension on HRQoL was carried out in northwestern Spain and found that the patients who were unaware of their hypertension reported a better subjective state of health than the patients who were aware of their hypertension.^11^ Another cross-sectional survey from Spain found that women who were aware of their hypertensive status reported worse HRQoL than those who were unaware for all SF-36 scales. The association was statistically significant for several SF-36 scales, bodily pain (*P* = 0.0129), general health (*P* = 0.0148), vitality (*P* = 0.027) and social functioning (*P* = 0.0198), and marginally significant for the role emotional scale (*P* = 0.0872).^29^ An observational study that used data from NHANES (2001–2004) also reported a significant adverse effect of hypertension awareness on HRQoL.^8^ Recently, a population survey in southwestern Finland (2005–2006) reported that the physical component of HRQoL is reduced in hypertensive patients who are aware of their condition compared with those who are unaware of their hypertensive status.^9^

The present cross-sectional study indicated a negative association between awareness of hypertension and HRQoL in hypertensive patients, but causality is not clear. Some studies have reported that the adverse association between awareness of hypertension and HRQoL is due to the chronic nature of the disease, medication effects, severity and duration of disease, or other social or psychological effects.^8,11^ In the present study, after adjusting for relevant socio-demographic, anthropometry, and lifestyle parameters, the variation of the *β*-estimates between awareness of hypertension and SF-36 was stable at most quantiles of the PCS and MCS scales. This result may indicate that these factors are not the primary explanations for the adverse effect of awareness of hypertension. Furthermore, adjustment for comorbidities led to a reduction of 40% in the magnitude of the association between hypertension awareness and HRQoL at all quantiles of the PCS scale. This result could at least partially explain why those who were aware of their hypertension were more likely to report poor PCS scores than those who were unaware. Comorbidity may be the cause of lower HRQoL in hypertensive patients who are aware of disease.

Another reason why hypertensive patients with awareness of their condition may display lower HRQoL is the adverse effects of antihypertension medication. In this study, awareness of hypertension, which was significantly associated with hypertensive patients’ PCS scores, had a great reduction of 42–90% in the HRQoL among those with poor PCS scale. This might indicate that antihypertension medication partially associated with the decreased in PCS scale. It is possible that people who require medication for their hypertension are more likely to have worse disease and, thus, more likely to have worse HRQoL than people who do not require medication and drugs.^29,32,33^ Additionally, because these data are from a cross-sectional study, we cannot assess how starting treatment, changing regimens, or stopping treatment may be related to the perceived HRQoL.

Our study suggests that comorbidities and adverse drug effects of anti-hypertension medication might be associated with deterioration of HRQoL. Thus, patients who had aware of hypertensive might have poorer HRQoL than those who were unaware. Health-care workers should not only control patients’ blood pressure but also pay more attention to taking care of patients’ physical and mental situation. Comprehensive health surveillance and psychological counseling could improve the HRQoL for patients who had aware of hypertensive. Furthermore, further studies should be carried out to explore whether hypertensive awareness itself may affect the Qol with sufficient medical, social, and emotional support.

### Strengths and Limitations

The major strength of the study was the use of QR. From the minimum to maximum response, this regression for modeling the PCS and MCS scores offers a more comprehensive picture of the relationship between variables than other regression models.^12,34,35^ Given that multiple quantiles can be modeled, it is possible to achieve a more complete and robust understanding of how the PCS and MCS score distributions are affected by awareness of hypertension, including information about changes in physical shape. Other strengths of our study include the large-scale recruitment of individuals living in rural areas, the completion of the SF-36 questionnaire filled before the diagnosis of hypertension, the exclusion of other chronic diseases that might reduce a patient's HRQoL, and the use of novel sensitivity statistical analysis. These measurements improved the validity and reliability of this study.

This study also had some limitations. First, the prevalence of hypertension was estimated according to a cross-sectional design, and it is difficult to establish the causal association even controlling for these cofounders. Second, the survey was mainly carried out at a single site, and selection bias might be present, which may restrict the application of the results to broader populations. Third, we did not use a specific questionnaire to measure side effects of blood pressure drugs or specific symptoms of blood pressure. Therefore, the influence of treatment of hypertension on quality of life is not clear. However, our findings have important implications for clinicians and researchers. Our application of QR models to skewed data enhances the validity of our results and offers a novel and relevant approach to the study of hypertension and HRQoL. The conclusion is similar to that of previous studies but more robust.

## CONCLUSION

In rural western China, awareness of hypertension was negatively related to HRQoL in hypertensive patients, which has a greater effect on MCS with the poorer status and on PCS with the intermediate and good status. Health-care workers should not only control patients’ blood pressure but also pay more attention to patients’ HRQOL. Measures are needed to improve the HRQOL of hypertensive patients.
